# Artificial intelligence-driven nano-enhanced stem cell therapy for neurodegenerative diseases: from rational design to clinical translation

**DOI:** 10.1186/s12951-026-04154-2

**Published:** 2026-02-19

**Authors:** Nan Chen, Shichan Wang, Jiyong Liu, Xiaoting Zheng, Huifang Shang

**Affiliations:** 1 Department of Neurology, Laboratory of Neurodegenerative Disorders, Rare Disease Center, West China Hospital, Sichuan University, Chengdu, 610041 People’s Republic of China; 2 West China School of Medicine, West China Hospital, Sichuan University, Chengdu, 610041 People’s Republic of China

**Keywords:** Artificial intelligence, Stem cell transplantation, Nanomaterials, Neurodegenerative diseases

## Abstract

Neurodegenerative diseases (NDs) are progressive and incurable central nervous system disorders characterized by the accumulation of pathological proteins and the loss of neurons. Although stem cell transplantation offers a new treatment option, its clinical application is severely hindered due to imprecise delivery, low survival rate, and undirected differentiation. Many studies have used nanomaterials to enhance stem cell therapy. However, the rational design of these multifunctional nanomaterials often requires a large number of experiments and calculations to determine the optimal parameters. Meanwhile, the diagnosis of NDs and the design of nanomaterials are being profoundly influenced by artificial intelligence (AI) and data-driven modeling. Based on these advancements, we propose that AI can guide personalized nano-enhanced stem cell therapies. This review explores how machine learning (ML) and deep learning (DL) can address the current challenges in stem cell therapy and nano-enhanced stem cell therapies. More importantly, it provides a systematic framework for integrating AI across the entire nano-enhanced stem cell therapy. We analyzed how AI can optimize the design of nanobiological materials, thereby enhancing the survival rate of stem cells, targeted delivery, directing differentiation, and controlling the release of loaded drugs. Additionally, we proposed that AI can be used for post-transplant tracking and prognosis management. Beyond summarizing parallel advancements, this review proposes a closed-loop system that integrates patient-specific data, AI-driven design, and real-time monitoring, aiming to advance truly personalized medicine for NDs.

**Graphical Abstract:**

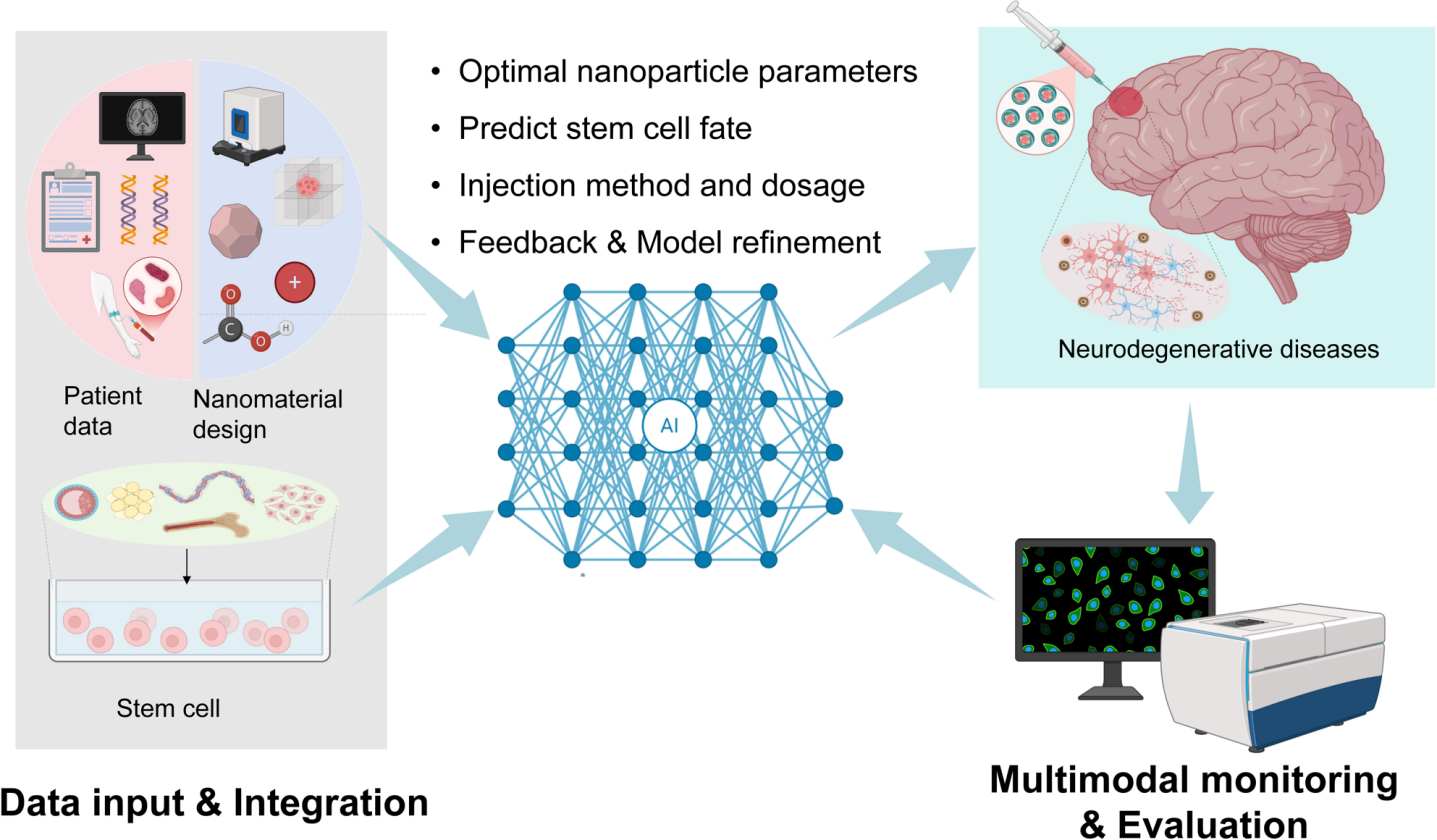

## Introduction

 NDs are progressive and incurable disorders of the central nervous system, characterized by the misfolding and aggregation of pathological proteins, neuroinflammation, and neuron loss [1, 2]. More than 70 million people have been suffering from Parkinson’s disease (PD), Alzheimer’s disease (AD), amyotrophic lateral sclerosis (ALS), and other neurodegenerative disorders so far [3–5]. As the population structure trend shifts towards aging, the prevalence of NDs is on the rise, imposing a huge burden on global health and the economy [6]. At present, the treatment of NDs mainly relies on drug therapy to control symptoms. However, there is no effective way to slow down the progression of the NDs, especially the rate of neuron loss.

Stem cells possess the ability to self-replicate and differentiate into multiple types, offering unprecedented possibilities for neuron regeneration. Therefore, researchers began attempting to treat NDs by transplanting stem cells over 20 years ago [7]. With the successful experimental results of stem cell transplantation in animal models for NDs, many clinical trials have begun to verify the therapeutic effects of stem cells [8–11]. As demonstrated by the promising direction shown in two important prospective clinical trials in *Nature*, transplanting human embryonic stem cells (ESCs) or induced pluripotent stem cells (iPSCs) into patients’ brains showed excellent safety, survival capabilities, and initial improvements in clinical symptoms [8, 9]. Critically, these grafts produced functional dopamine, evidenced by a putaminal 18 F-DOPA uptake increase of up to 44.7%. Meanwhile, the high-dose ESC cohort achieved an average improvement of 23.0 points in the MDS-UPDRS Part III score. However, broader clinical trials reveal persistent challenges that limit the widespread application of stem cell transplantation. Firstly, therapeutic efficacy remains dose-dependent and transient. In a PD trial (NCT04928287), benefits from mesenchymal stem cells (MSCs) were lost by 52 weeks. A large ALS trial found no significant treatment effect (NCT03280056). Secondly, safety concerns are notable, including high rates of fever (up to 100%) after intraventricular MSC delivery (NCT02054208) and serious adverse events like pulmonary embolism (NCT03117738). Thirdly, technical barriers remain substantial, with frequent cell mis-localization (NCT02943850). In addition, many stem cell transplants have adopted long-term immunosuppressive regimens, which bring additional risks such as infections and liver/kidney toxicity. For elderly and frail patients, this treatment poses a high risk. In brief, achieving the optimal and individualized cell delivery remains a challenge.

Nanomaterials are substances within the range of 1 to 100 nm. Their unique physical and chemical properties have been innovatively applied in the clinical transformation of stem cells to treat neurological diseases [12]. Engineering stem cells with nanotechnology provides spatiotemporal control over cellular functions [13]. Designing functionalized nanomaterials such as hydrogels, liposomes, and exosomes makes the carrying of antioxidants and neurotrophic factors possible [14, 15]. This creates a supportive niche for stem cells, inhibiting cell apoptosis and promoting survival and differentiation. Moreover, stem cells loaded with magnetic nanomaterials can be guided by an external magnet to migrate directionally to the brain region, thereby improving delivery efficiency [16–18]. The special physical properties of the nanosized-loaded stem cells, such as magnetism or photosensitivity, can establish connections with the outside world. After the stem cells are transplanted into the body, the connections enable the directed differentiation into astrocytes, functional neurons, and oligodendrocytes [19, 20]. The connections between nanomaterials and the outside world can also be utilized to achieve non-invasive spatial tracking and functional assessment of stem cells [21–23].

However, engineering an effective nano-enhanced stem cell therapy presents a complex rational design problem. The therapeutic outcome depends on a high-dimensional parameter space encompassing nanomaterial properties (size, shape, composition, surface charge), biological interactions (cell type, ligand density), and responses to exogenous stimuli. Critically, the relationships between these parameters and the outcomes (cell survival, targeted homing, and controlled differentiation) are often non-linear and non-intuitive. Traditional trial-and-error experimentation is unable to navigate this vast combinatorial data efficiently. This fundamental challenge, coupled with the inability to achieve dynamic and post-transplant prognosis management, creates a compelling need for intelligent methods. This is where artificial intelligence (AI), primarily through its branches of ML and DL, has the potential to make a difference [24]. ML is typically employed to model structured parameters and predict optimal designs, whereas DL excels at analyzing complex patterns in imaging and omics data [25, 26]. AI is based on data computation as its core method, and utilizes machines or programs to simulate human learning, reasoning, perception, and decision-making. It can analyze vast amounts of data from previous experiments to predict the biological properties of new nanomaterials [27]. After training, AI can also reverse-engineer from the expected therapeutic effects to propose the optimal parameters of nanomaterials. By integrating clinical information of patients with NDs into an AI-based platform and using computer simulation design, nanocarried stem cells can be adapted to the unique disease states of individuals, thereby achieving the maximum efficacy of personalized treatment [28].

This review proposes that converging AI, nanotechnology, and stem cell transplantation creates a new frontier in NDs treatment. Through synthesizing the current hurdles in nano-engineered stem cells, we stress the necessity of incorporating AI into this treatment. Crucially, we focus on how AI tackles nanomaterial design, stem cell-controlled differentiation, and post-transplant monitoring. By providing a comprehensive overview and a critical perspective, this review aims to serve as a roadmap for researchers at the intersection of AI, nanomedicine, and stem cell biology.

## Stem cell therapy and persistent hurdles

The concept of using stem cells to treat NDs was first proposed in the 1990 s [29]. Over the past 30 years, MSCs, neural stem cells (NSCs), iPSCs, and ESCs have been applied in several clinical trials, bringing hope for the treatment of ALS, PD, and AD (Table 1). Although the exact mechanism is unclear, the results of clinical trials indicate that these stem cells have neuroprotective effects, delay the progression of NDs, and even prolong life. In animal models, stem cell transplantation can prevent the further loss of neurons during disease [30, 31]. However, the low neuronal differentiation ability and survival rate after transplantation limit the efficacy of stem cells [31–33].

**Table 1:** Different stem cell types for neurodegenerative disease therapy

Cell types	Primary sources	Therapeutic mechanism	Advantage and Key trials	Challenges and Key trials
MSCs	Bone marrow, adipose tissue, umbilical cord, etc.	Paracrine signaling (neurotrophic factors, exosomes) [34, 35], immunomodulation [36], and anti-inflammation [37].	NCT04928287 (PD, Phase II, *n* = 24, intravenous injection): A mild improvement in MDS-UPDRS II (−2.07 points) at Week 8 NCT02054208 (AD, Phase I/IIa, *n* = 46, intraventricular injection): Technically feasible and safe [38]. A rapid but transient modulation of CSF biomarkers NCT04821479 (ALS, Phase I/II, *n* = 20, intrathecal injection): 58% progression rate slowed per month [39] NCT03828123 (ALS, Phase I/II, *n* = 26, intrathecal injection): 80% patients maintained stable lung function (Forced Vital Capacity ≥ 70%) for 9 months. 75% patients showed stable limb strength (weakness score change < 1.0) for 3 months [39]	NCT04928287 (PD, Phase II, *n* = 24, intravenous injection): The benefit was lost by Week 52 NCT04821479 (ALS, Phase I/II, *n* = 20, intrathecal injection): Efficacy waned with repeated dosing [39] NCT03280056 (ALS, Phase I/II, *n* = 196, intrathecal injection): No statistically significant differences NCT03117738 (AD, Phase I/II, *n* = 21, intravenous injection): A deterioration in the ADAS-Cog (+2.9 points). Three serious adverse events, including pulmonary embolism NCT02054208 (AD, Phase I/IIa, *n* = 46, intraventricular injection): 100% fever [38] NCT03172117 (AD, Phase I/IIa, *n* = 36, intraventricular injection): 87.5% fever
NSCs	Fetal tissue, differentiation from hiPSCs, etc.	Cell replacement, neurotrophic support, and promotion of myelination [40, 41]	NCT02943850 (ALS, Phase I/IIa, *n* = 18, unilateral lumbar spinal cord injection): Transplanted cells survived and continuously secreted GDNF for up to 42 months [11]. No serious adverse events	NCT02943850 (ALS, Phase I/IIa, *n* = 18, unilateral lumbar spinal cord injection): Most cells localized to the dorsal (sensory) horn, not the target ventral (motor) horn. At autopsy, benign neuromas (1–3 mm) were detected at injection sites [11]
iPSCs	Somatic cell reprogramming	Cell replacement, disease modeling [42, 43], and personalized therapy [44]	No efficacy data have been published yet from interventional trials	No efficacy data have been published yet from interventional trials Complex and costly manufacture
ESCs	Inner cell mass of the blastocyst	Cell replacement	NCT03482050 (ALS, Phase I/IIa, *n* = 16, intrathecal injection):53% ALSFRS-R decline slowed in the first 3 months [45]	NCT03482050 (ALS, Phase I/IIa, *n* = 16, intrathecal injection): no healthy controls [45] Requires immunosuppression Embryonic origin poses ongoing ethical challenges

### Survival dilemma

As early as 2007, Ute Schafer et al. discovered that the inflammatory response would damage the survival of stem cells in mouse models, characterized by macrophage accumulation, astrocyte proliferation, and microglia activation in the brain [46]. Similar correlations were also observed in the ALS mouse model following cervical spinal cord transplantation of stem cells. Only about 28.3% of transplanted NSCs survived at 4 weeks post-transplantation, with complete loss by 8 weeks. This poor survival was linked to a hostile microenvironment, characterized by mature macrophages and marked gliosis [47]. Importantly, these observable cellular changes are probably driven by inflammasome activation and metabolic stress. Specifically, NLRP3 inflammasome activation triggers pyroptosis and the release of IL-1β, directly damaging stem cells [48, 49]. Concurrently, local hypoxia and nutrient deprivation critically impair stem cell function and survival within the diseased parenchyma [50, 51]. While intracerebral stem cell injections have been explored for treating NDs, this invasive method itself can induce post-injury inflammation, further activating these detrimental pathways and reducing stem cell viability [52, 53].

### Delivery and retention challenges

Non-invasive intravenous infusion shows poor central nervous system targeting and transient effects. A Phase II trial of PD (NCT04928287) reported a mild and short-term improvement by Week 8, which was completely lost by Week 52. More concerningly, a trial for AD (NCT03117738) resulted in clinical decline and serious adverse events. Invasive CSF-space delivery improves initial access but contends with waning efficacy, variable outcomes, and frequent inflammatory side effects. For instance, intraventricular injection in AD (NCT02054208) was feasible and induced rapid CSF biomarker modulation, yet effects were transient and accompanied by a universal incidence of fever (100%) [38]. In ALS, intrathecal injection (NCT04821479) demonstrated a significant slowing of progression, but benefits were noted to wane with repeated dosing [39]. Stereotactic intracerebral injection enables durable local engraftment but faces hurdles in targeting accuracy. A trial for ALS (NCT02943850) confirmed the potential for long-term survival and sustained secretion of the therapeutic factor GDNF for over 3 years [11]. However, post-mortem analysis revealed that the majority of transplanted cells localized to incorrect (sensory) regions of the spinal cord rather than the target (motor) area, and benign neuromas were found at injection sites. These highlight persistent challenges in surgical precision, accurate cell distribution, and long-term safety monitoring.

### Uncontrolled differentiation and tumorigenic risks

In the MPTP-induced Chlorocebus sabaeus model, after implanting human NSCs into the caudate nucleus and substantia nigra, more than 80% of the human NSCs migrated from the implantation site to the damaged striatal pathways. However, the migrated stem cells had a glial lineage rather than a neuronal lineage, and over 90% human NSCs maintained an undifferentiated and quiescent progenitor cell state [54]. Apart from the unsatisfactory differentiation direction, stem cells may remain in a quiescent state and fail to differentiate after transplantation [31–33]. Furthermore, the transplantation of multipotent ESCs carries the risk of tumor formation, especially teratomas [55].

**Fig. 1:**
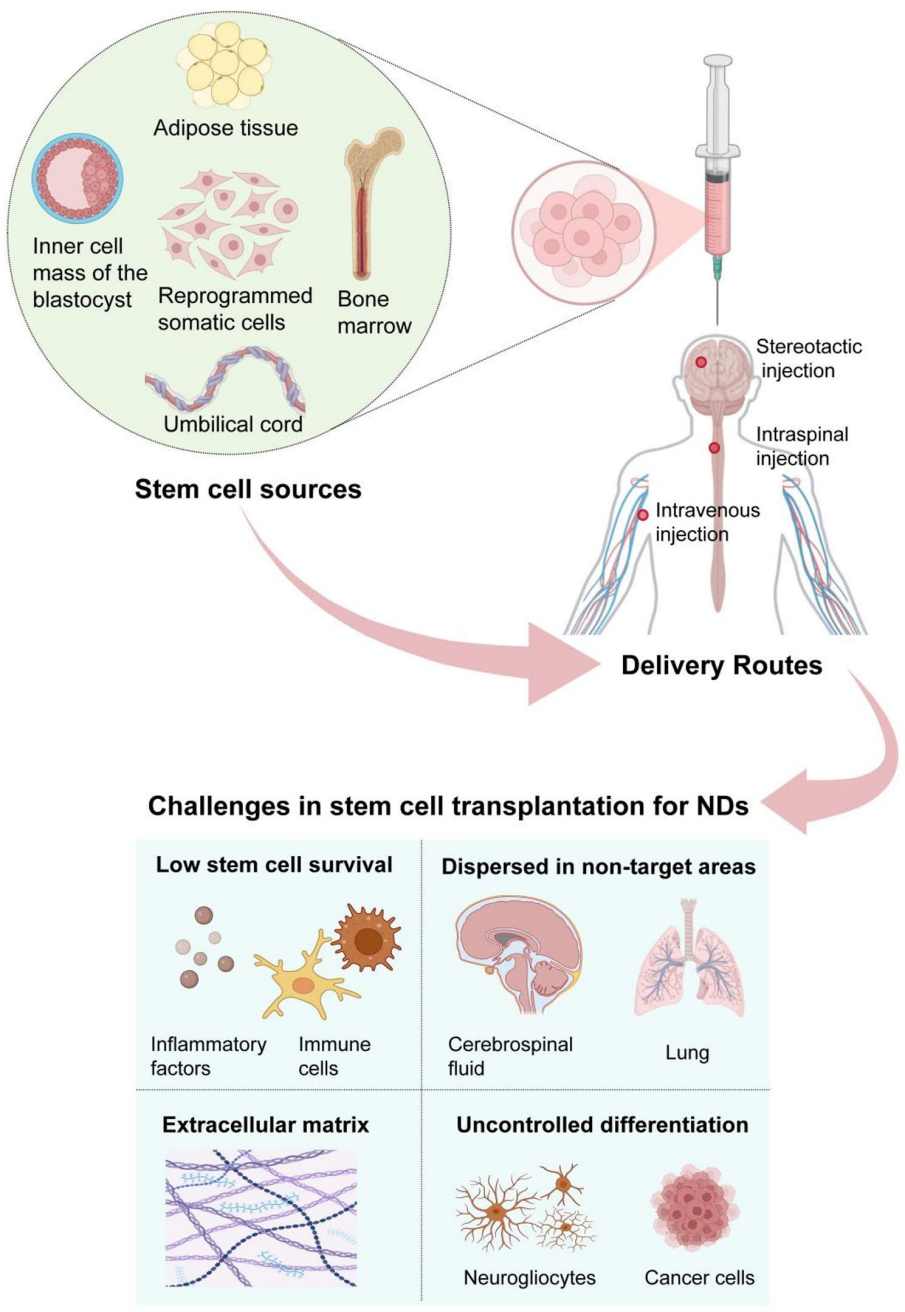
The main challenges in stem cell therapy for NDs. Adipose tissue, reprogrammed somatic cells, inner cell mass of the blastocyst, bone marrow, umbilical cord, etc., are the main sources of stem cells. Stem cells can be injected into patients through stereotactic injection, intraspinal injection, and intravenous injection. However, this process faces four primary challenges: (1) low stem cell survival at the target site, (2) dispersion to non-targeted areas, (3) the inhibitory role of a pathologically altered extracellular matrix, and (4) the risk of uncontrolled differentiation and potential tumor formation

In conclusion, the clinical translation of stem cell therapy in NDs faces three major challenges (Fig. 1). Firstly, the hostile immune microenvironment of the host after stem cell transplantation severely impairs cell survival. Secondly, the non-targeted delivery methods and low retention rate in the lesion site result in limited therapeutic effects. Thirdly, the uncontrollable differentiation fate may lead to treatment failure or trigger tumorigenic side effects. These problems indicate that the traditional stem cell transplantation strategies are approaching a bottleneck, which has given rise to the nanomaterial-engineered stem cell technology.

## Nanomaterial engineering: armoring stem cells to improve therapeutic effects

During the process of solving the conundrums above, researchers gradually realized that nanomaterials stand out due to their unique size effect, modifiable physical and chemical properties, and good biocompatibility. As described in this section, by combining stem cells with functionalized nanomaterials, we can not only protect stem cells from inflammatory attacks but also achieve precise delivery and directed differentiation of stem cells [56].

### Enhancing survival

The traditional suspension injection fails to provide physical anchoring and protection for stem cells, which may contribute to the extremely low survival rate in vivo. As a biomimetic 3D material, hydrogels have been widely studied to improve this situation. The efficacy of hydrogels critically depends on their characterized physicochemical properties, such as mechanical modulus and network structure, which govern cell support. Grosskopf et al. designed an injectable polymer nanoparticle hydrogel for human MSCs, quantifying its shear-thinning behavior, self-healing kinetics, and yield stress (e.g., ~ 60 Pa for the 1:5 formulation). Their experimental results in immunocompetent mouse models have demonstrated that PNP hydrogel could significantly enhance the retention of human MSCs at the injection site. By the 14th day, cell retention was 40 times higher than that of the traditional PBS injection [57]. In addition, Xu et al. developed a bioactive self-healing hydrogel based on an oxidized tannic acid modified gold nano-crosslinker, reporting a storage modulus of ~180 Pa and a porous structure (average pore size ~59.7 μm) suitable for neural applications. This hydrogel could also remain in the target brain region for more than 14 days. By polarizing the pro-inflammatory M1 type microglia into anti-inflammatory M2 type and eliminating approximately 90% of reactive oxygen species (ROS), the survival rate of dopaminergic neurons in the substantia nigra-striatum region was increased, and the α-syn aggregation level decreased by ^58^. Cell membrane biomimetic technology is another innovative therapy to address the low survival rate of stem cells. Chen et al. extracted the stem cell membranes from rat bone marrow and then encapsulated them on the surface of mesoporous polydopamine [59]. This method utilizes the functional proteins on the stem cell membrane to cross the blood-brain barrier (BBB) and arrive at the lesion site. When attacked by the immune system, the cell membrane ruptures and mPDA inside is released, effectively clearing the ROS in the lesion area like a Trojan horse.

### Enabling targeted delivery and retention

Recruiting endogenous NSCs to the lesion site can enhance the regenerative potential to a certain extent. However, the traditional chemokine recruitment method lacks specificity and may lead to unnecessary inflammatory or fibrotic reactions. Sun et al. designed a nanoparticle carrying a selected antisense oligonucleotide. To mediate the recruitment of endogenous NSCs, he coated the nanoparticles with NSCs membranes and linked the membranes with the aptamer Apt 19 S. The particles facilitated the penetration of the nanoparticle through the BBB and promoted the recruitment of NSCs to the PD site for dopaminergic neuron regeneration [60]. Although intracerebroventricular injection can deliver more stem cells into the brain compared to intravenous infusion, most of the injected cells will be lost through circulation in the CSF, resulting in a low retention rate in the brain. In Jung’s research, superparamagnetic iron oxide (SPIO) nanoparticles were internalized into MSCs, thereby allowing stem cells to be captured by magnetic force in vitro model simulating the flow of CSF [16]. However, the internalization of SPIO nanoparticles may induce iron overload and elevated ROS generation, potentially compromising MSC viability and function [61, 62]. These risks can be mitigated through using lower molecular weight polycation coatings like PEI2k and optimizing the SPIO/polymer ratio, thereby reducing cytotoxicity without sacrificing labeling efficiency or MRI sensitivity [62]. Thus, evaluating potential cytotoxicity is essential for the safe and effective use of SPIO-MSCs. To target the highly expressed vascular cell adhesion molecule 1 (VCAM-1) on the inflammatory blood vessels in the brain of AD patients, the researchers combined biomimetic 4 F peptides containing disc-shaped high-density lipoprotein with the VCAM-1 VBP peptide. Then they co-incubated the material with MSCs, engineering stem cell membranes via lipid raft-mediated embedding of the VCAM-1-targeting VBP peptide. This modification enables them to precisely recognize and adhere to the inflammatory cerebral endothelium, thereby facilitating efficient receptor-mediated traversal of the BBB [63]. These engineered MSCs demonstrated a high enrichment rate and long-term retention ability in the brains of AD model mice, effectively improving neuronal function and cognitive impairment [63]. The precision of targeting is governed not only by ligand specificity but also by its surface ligand density. This parameter non-linearly influences receptor binding kinetics, clustering, and downstream intracellular signaling. For instance, in biomaterial scaffolds, varying the density of immobilized peptides on microgel surfaces directly dictates the spreading, proliferation, and differentiation of MSCs [64]. Translating this to neural targeting, the density of peptides targeting the BBB or neuronal adhesion molecules must be finely optimized to achieve the balance between homing and functional integration of nano-engineered stem cells.

Collectively, magnetic guidance provides spatially precise and externally controlled targeting. It enriches cells at a predetermined lesion site regardless of the local cellular phenotype, and excels in overcoming CSF clearance and achieving high local concentration. Conversely, aptamer-based or peptide-based molecular guidance relies on the affinity of ligands for receptors on specific pathological cells or endothelia. This enables homing to a defined zone, potentially minimizing off-target engagement in healthy tissue adjacent to the lesion. Deconstructing and optimizing the parameters of such hybrid strategies presents a quintessential challenge for AI-driven design.

### Guiding differentiation and fate

Precisely controlling the differentiation direction of stem cells in vivo is a key challenge in neuroregeneration therapy, especially overcoming their inherent tendency to differentiate into glial cells rather than neurons. Owing to a complex intracellular microenvironment, simple delivery of growth factors often has limited effects. Zhang et al. designed a type of nanoparticle that simultaneously delivers retinoic acid (RA) and small interfering RNA targeting SOX9(siSOX9) protein, and encapsulates SPIO for tracking the nanoparticle. SOX9 is a key protein that inhibits the expression of neuronal genes and induces glial characteristics, and RA can upregulate neuronal-related genes. The nanoparticle-to-siRNA (N/P) ratio was optimized to 5:1, ensuring complete siSOX9 complexation, a stable nanoparticle size (100 nm), and high cell viability (> 92%). Besides, RA loading reached 87 µg/mg nanoparticles. The design features a critical temporal release profile: siSOX9 is released first in endosomes/lysosomes, achieving 52.3% SOX9 mRNA knockdown, followed by the sustained and esterase-dependent release of RA (60% within 1 day). The downregulation of SOX9 and the upregulation of RA increase the efficiency of NSCs to differentiate into neurons from 11.1% to 76.8%. After transplanting these nano-engineered stem cells, not only can the memory function of AD model mice be significantly improved and neuronal damage be repaired, but also the encapsulated SPIO nanoparticles can achieve real-time magnetic resonance imaging (MRI) tracking for up to 35 days [65]. This truly represents a new diagnosis and treatment model that integrates treatment and monitoring. Based on this, Yu et al. integrated cerium dioxide nanoenzymes with superoxide dismutase and catalase activities into the metal-organic frameworks carrier [66]. This enabled the differentiation of NSCs to be induced while also protecting the newly generated neurons by eliminating ROS.

In recent years, manipulating the physical microenvironment of cells to regulate their biological behaviors has become an emerging strategy. Zhang et al. developed a multi-layer core-shell structure of upconversion nanoparticles (UCNPs) that responds to 808 nm near-infrared (NIR) light. The particle can efficiently convert the highly penetrating 808 nm NIR light into local ultraviolet emission, thereby unlocking the “gate” of the mesoporous silica shell and releasing the loaded neural differentiation factors. Once NIR irradiation stops, the polymer gating returns to its hydrophobic state, and the release immediately ceases [67]. This reversible “on-off” release characteristic enables researchers to remotely control the neuronal differentiation of human iPSC-derived NSCs with unprecedented precision. Apart from biochemical signals and optogenetic methods, biological electrical signals can also be used to regulate cell fate. However, traditional electrical stimulation methods rely on invasive electrodes. The emergence of piezoelectric nanomaterials provides a new solution for non-invasively simulating endogenous electric fields. James et al. integrated titanium dioxide barium oxide piezoelectric nanoparticles into 3D human NSCs spheroids, and uniform electrical stimulation was achieved throughout the entire 3D sphere by utilizing poly-D-lysine and low-frequency ultrasound (40 kHz). No significant adverse effects on cell viability or membrane integrity were observed following the stimulation protocol. Applying ultrasound-mediated piezoelectricity during the differentiation stage could guide human NSCs to differentiate into the neuronal lineage and enhance the synaptic formation ability of neurons [68].

### Facilitating monitoring and feedback

Long-term, non-invasive, and high-resolution in vivo tracing is crucial for evaluating the efficacy of stem cell therapy. The choice of imaging modality is dictated by the specific biological question, as each technique presents a unique compromise between spatial resolution, sensitivity, penetration depth, and temporal dynamics. For anatomical localization with excellent spatial resolution (typically 50–200 μm), Betzer et al. developed a gold nanoparticle-based CT imaging technique to track transplanted human MSCs for up to one month [69]. In contrast, MRI provides superior soft-tissue contrast and high sensitivity to iron-based contrast agents at the nano-molar level, enabling long-term tracking. Liu et al. encapsulated SPIO nanoclusters with low-molecular-weight amphiphilic polymer N-alkyl-PEI2k for labeling and tracking of MSCs. Without affecting the survival, proliferation, and differentiation of stem cells, the labeled MSCs transplanted under the skin of mice could be monitored for up to 19 days with an MRI detection limit of ~2 µg Fe/mL [62]. Clay et al. ingeniously utilized the fluid shear force generated by the orbital shaker as a physical stimulus to increase the SPIO uptake efficiency of MSCs by nearly twice [70]. However, this method also introduces new optimization variables, such as the optimal shear force magnitude, the duration of action, and compatibility with different cell types. Furthermore, Chen et al. combined multimodal imaging with nanomedicine to conduct in situ tracking and long-term monitoring of stem cells [71]. The researchers utilized multifunctional nanoseaurchin probes to simultaneously provide both photoacoustic imaging (PAI) and MRI data, and successfully introduced them into stem cells. PAI offers high optical sensitivity and real-time imaging capability at moderate spatial resolution (tens to hundreds of microns), which was used for short-term monitoring of magnetically guided stem cell homing. MRI, with its whole-body penetration and high soft-tissue resolution, was then used for long-term tracking of cell survival and distribution. Beyond demonstrating excellent imaging performance, this study provided direct evidence that the labeled stem cells retained their therapeutic paracrine function. Upregulated anti-inflammatory cytokines (IL-10, TGF-β) and downregulated pro-inflammatory factors (IL-1β, IL-6, TNF-α) were observed. After combining multimodal imaging with loaded nanocells, the infarct volume decreased, and neurological function recovered in stroke mice. In the future, such abundant multimodal imaging data can be perfectly used as input for training AI models. Beyond tracking cellular location, nanomaterials can also be designed as ultra-sensitive biosensors to monitor stem cell function and differentiation. Rabie et al. constructed a biosensor using core-shell-shell UCNPs, achieving picomolar-level sensitivity for dopamine detection. This represents an extreme in sensitivity, orders of magnitude higher than typical MRI or PAI contrast thresholds, enabling non-destructive detection of trace neurotransmitters released from individual stem cell-derived neurons [72]. However, this ultra-high sensitivity comes at the cost of very limited penetration depth (typically sub-millimeter), confining its application to superficial or in vitro settings. In summary, while CT provides hard-tissue anatomy, MRI offers deep-tissue, long-term cell tracking, PAI enables real-time vascular and functional imaging, and UCNP-based fluorescence achieves unparalleled molecular sensitivity at shallow depths, the optimal strategy often involves their complementary use.

Beyond therapeutic functions, the fate of nanomaterials within the body is another paramount safety consideration. Biodegradation timelines vary dramatically across nanomaterial classes. Polymeric carriers like lactic-co-glycolic acid or chitosan degrade over weeks to months via hydrolysis, with rates tunable by molecular weight and crystallinity [73, 74]. Inorganic nanoparticles undergo slower, metabolically-driven dissolution or persist, while hydrogels degrade based on cross-linking density and enzymatic activity [75]. This temporal mismatch between therapeutic need and material persistence necessitates precise design. More critically, degradation products can directly modulate neuroinflammation in complex ways. A notable example is a transferrin receptor-targeted mesoporous nanoselenium carrier Met@MSe@Tf, which dissociates in the acidic environment of AD lesions and thus releases metformin. This degradation enhances the phagocytic clearance of Aβ plaques by microglia, and alleviates neuroinflammation and oxidative stress [76]. Similarly, nanoparticles designed to degrade formaldehyde or bilirubin have shown efficacy in reducing neuroinflammation and associated damage in depressive and hyperbilirubinemia models [77, 78].

In conclusion, nanomaterials enhance the efficiency of stem cell therapy by constructing biomimetic microenvironments, achieving targeted delivery, mediating precise drug or gene delivery, and enabling real-time imaging.

However, designing nano-engineered stem cell therapies faces several challenges. Firstly, the design and synthesis of nanomaterials involve complex, multi-parameter optimization. These parameters encompass material properties, biological interactions, and responses to exogenous stimuli (Table 2). Secondly, the process is further complicated by batch-to-batch variability during nanomaterial synthesis and nonlinear interactions between materials and the in vivo biological environment. Traditionally, researchers have addressed these optimization problems through sequential and low-throughput experimentation. This strategy is not only prohibitively slow and resource-intensive, but also difficult to capture the underlying patterns within such a complex system.

Therefore, moving from empirical, trial-and-error approaches to a predictive, data-driven design framework is imperative. The need to extract optimal solutions from massive, multidimensional datasets constitutes the core rationale for the integration of AI. AI, with its capacity for pattern recognition, modeling of nonlinear relationships, and iterative optimization, is uniquely positioned to solve these bottlenecks.

**Table 2:** Summary of critical parameters for nano-engineered stem cell therapies and their role as feature inputs for AI model development

Parameter category		Design element	Function	Refs
Material properties	Size	1–100 nm	Endocytosis pathway and distribution in vivo.	[34, 79]
	Shape	Spherical, rod-shaped, sheet-shaped, star-shaped, etc.	Cell uptake efficiency, endocytosis pathway, and in vivo retention rate	[79, 80]
	Composition	Polymers (PLGA, PEG), liposomes, inorganic materials (gold, silica, iron oxide), carbon materials, etc.	Different biocompatibility, degradability, and functional properties	[81]
	Surface charge	Positive, neutral, negative	Bind with the negatively charged cell membrane, promote endocytosis, reduce non-specific binding, and prolong the retention time in vivo	[82]
	Surface functional groups	-COOH, -NH2, -SH, etc.	Stability, protein adsorption, and interaction with cell membranes	[83, 84]
	Stiffness/Elastic modulus	Soft, stiff.	Simulate the in vivo microenvironment to provide mechanical signals to stem cells, regulating their differentiation and function	[85]
	Degradation rate and product	Controllable, non-toxic metabolites	Matched with the therapeutic time and purpose, non-toxic and metabolizable	[86, 87]
Biological interactions	Stem cell type	MSCs, NSCs, iPSCs, ESCs	Different stemness, paracrine signature, and differentiation potential	[34, 88]
	Surface ligand density	Number of targeting moieties per unit area	Regulates receptor binding affinity, cellular signaling, and fate. Optimal density balances multivalent engagement with steric hindrance	[64]
	Loading/Combination strategy	Internalization/incubation, surface-anchoring/chemical conjugation	Integrate with stem cells, secrete chemical factors	[89, 90]
	Delivery conditions	Incubation environment, time, and environment	Ensure effective combination or internalization of nanomaterials with stem cells	[91]
	Hydrogel matrix properties	Stiffness, degradation rate, ligand density, porosity, and viscoelasticity	3D support mimicking extracellular matrix, spatiotemporal presentation of biochemical and mechanical cues, and enhance retention at the transplantation site	[92–94]
Exogenous stimuli	Magnetism	SPIO, etc. Field Strength/Gradient	Targeted homing via external magnetic field guidance	[81, 95, 96]
	Electrics	Piezoelectric materials, etc. Field Strength/Frequency	Stimulate differentiation, mimic native bioelectricity	[97]
	Light	Gold nanorods/shells, UCNPs, etc. Wavelength/Intensity/Pulse Duration	Remote control of ion channels/cell signaling	[21, 98]
	Ultrasound	Frequency/Intensity	Enhance cell membrane permeability for improved cargo delivery, trigger drug release from nanocarriers, and imaging guidance	[21]

## AI revolution: intelligently designing and optimizing nano-engineered stem cell therapies

**Fig. 2:**
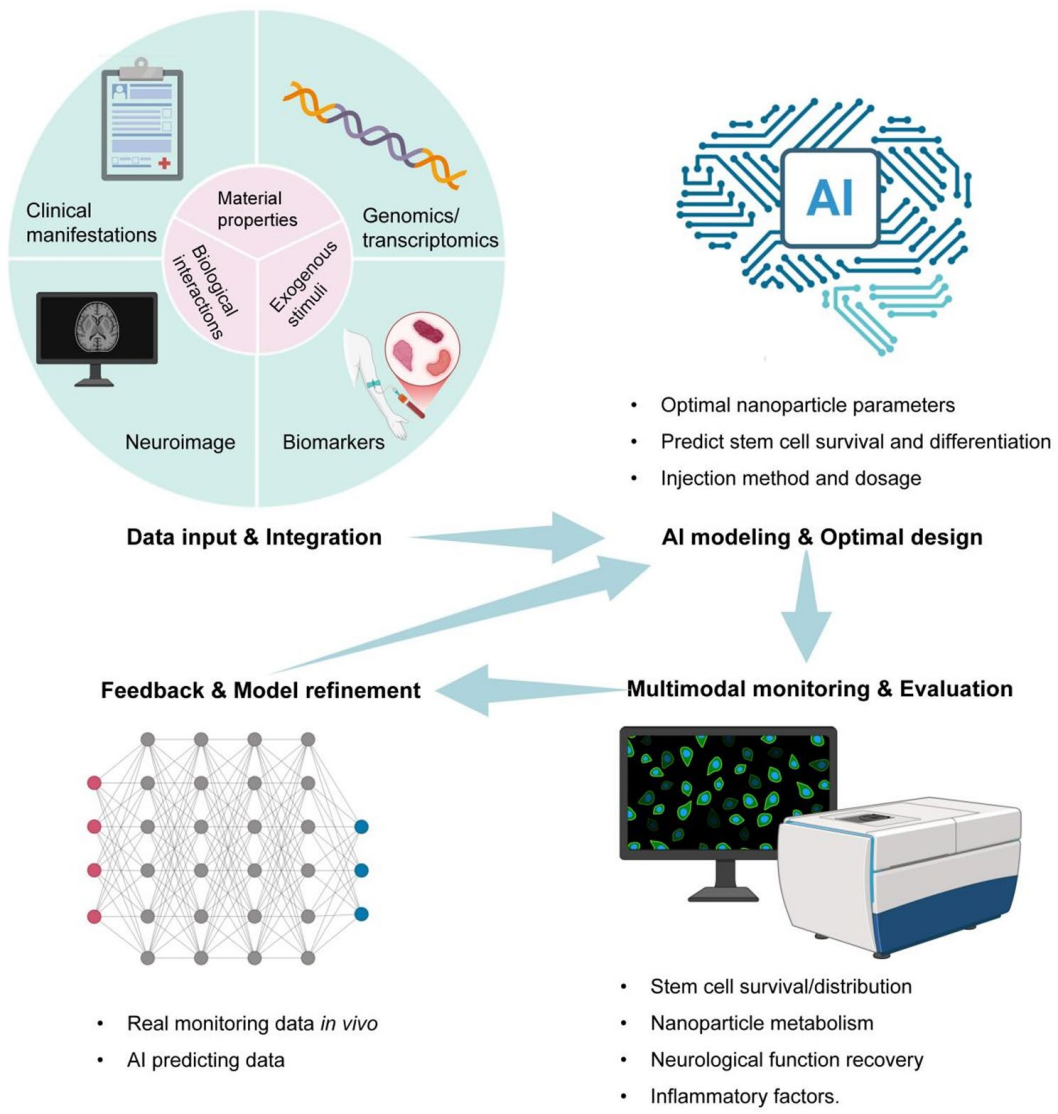
Conceptual framework of the closed-loop and AI-driven personalized therapy platform. This figure illustrates the proposed iterative workflow for designing and optimizing nano-enhanced stem cell therapies for NDs. The cycle begins with the Data Input & Integration phase (top-left), where patient-specific multimodal data (clinical manifestations, genomics/transcriptomics, neuroimaging, and biomarkers) is fused with the parameters of nano-stem cell constructs (material properties, biological interactions, and exogenous stimuli). This integrated dataset feeds into the AI Modeling & Optimal Design module (top-right), where machine learning models perform multi-objective optimization to predict key outcomes, such as optimal nanoparticle parameters, stem cell survival/differentiation efficiency, and personalized injection method/dosage. Following clinical administration, the Multimodal Monitoring & Evaluation module (bottom-right) tracks real-time in vivo responses, including nanoparticle metabolism, neurological function recovery, and inflammatory factor dynamics. Crucially, this real-world monitoring data is compared against initial AI predictions in the Feedback & Model Refinement phase (bottom-left). Discrepancies and new insights are used to continuously retrain and refine the AI models, closing the loop

As the requirements for the properties and accuracy of nanomaterials continue to increase, the design and optimization require quite a lot of computational expense and time. Finding the best solution among numerous parameters to improve nano-engineered stem cell therapy in NDs is a major challenge. This is exacerbated by the fact that data generated from traditional optimization may contain biases from specific synthesis batches. By analyzing high-dimensional data, AI can utilize complex computational methods to learn and apply relevant parameters from complex data sets, thereby accurately designing optimal nanomaterials for loading stem cells and accelerating the development of personalized therapies (Fig. 2). Therefore, combining AI with nanotechnology is an innovative development trend.

However, it is crucial to emphasize that the majority of AI applications discussed in this section are currently at the proof-of-concept stage in controlled environments. Translating parameter optimization into clinically viable nano-enhanced stem cell therapy constitutes a major and unresolved technical challenge. The following examples illustrate promising directions, but their translation requires overcoming multi-parameter modeling and integration.

### Nanomaterial design

In the field of nanomaterial design, the application of AI has significantly enhanced the ability to predict and control the complex behaviors of nanobiological interfaces. Johnston et al. developed an interpretable ML method for simulating the binding dynamics of nanoparticles and cells based on experimental data [99]. They used neural networks to identify the model of nanoparticle-cell interactions and provide quantitative parameters. This work facilitates the quantitative design of nanomaterials and predicts the interaction between stem cells and nanomaterials. Microfluidics is an efficient method for controlling the size and uniform distribution of nanoparticles. Precisely controlling the physical and chemical properties of nanoparticles using microfluidic parameters requires a significant amount of computation and experience. Smeraldo et al. developed an artificial neural network (ANN) model to predict the size and distribution of nanoparticles [100]. Based on the flow rate and polymer concentration of microfluidics, the model achieved a prediction accuracy of up to 98.9% (R² = 0.9734) (Table 3).

When nanoparticles enter the culture medium or body fluids, proteins and other biological molecules will adsorb onto the surface, forming a protein corona. This significantly alters the physical and functional properties of the nanoparticles. Traditionally, it was believed that this process was chaotic and unpredictable, but recent studies have shown that ML can interpret and predict the rules governing the formation of the corona layer [101]. Ban et al. employed ML, particularly the random forest (RF) algorithm, to accurately predict the functional composition of the protein corona formed by nanoparticles in body fluids [102]. Most importantly, this study directly linked the predicted functional corona with the fate of the loaded cells, with high predictive accuracy (R² > 0.75 for most functional protein classes) (Table 3). This means that in the design process of nanosystems for cell loading, there is no need to synthesize nanoparticles first and then expect to obtain the desired charge layer. Instead, we can utilize such ML models to reverse-engineer the surface chemical properties of nanoparticles, forming the required charge distribution. For example, we can design a surface rich in anti-phagocytic proteins, such as apolipoproteins and clusterin in the previous predictive model [102], to evade immune clearance and prolong the circulation time of nano-engineered stem cells. Additionally, by inputting the characteristics of the nanomaterials used to activate stem cells, we can predict their performance in complex CSF or brain parenchyma, reducing the risk of tumor formation. Nevertheless, the predictive accuracy of these models in clinical applications may be influenced by patient-specific variables. As highlighted in a recent review, factors such as biological sex, genetics, age, environmental exposures, and disease state alter the composition of biological fluids [103]. This variability impacts the protein corona, potentially affecting the model predictability. Consequently, while the model proposed by Ban et al. provides a powerful framework, its translation to personalized NDs nanotherapy strategies requires further optimization for individual fluid variations.

This model can be further optimized to personalize the unique plasma and CSF of patients, thus achieving personalized nanotherapy strategies for NDs. To gain a more fundamental understanding of the interactions among nanomaterials, body fluids, and stem cells, researchers have turned their attention to underlying physicochemical parameters. Sengottiyan eta al. focused on zeta potential (ζ), a nanoparticle parameter that controls the stability, aggregation, and initial attachment with stem cells [104]. However, predicting ζ is challenging because the protein corona masks the original surface charge dynamically. To solve this problem, they combined the genetic algorithm with partial least squares regression, developing a predictive nano-quantitative structure property relationship model. This model can quantitatively analyze the core, coating of nanoparticles and the influence of the protein corona on the final ζ.

### Stem cell manipulation

In the process of stem cell preparation and quality control, traditional screening methods such as flow cytometry and immunocytochemistry are usually inefficient, costly, and destructive. AI-driven image recognition technology is revolutionizing the traditional methods that rely on manual marking to suit large-scale clinical production. Beuckeleer et al. combined cell painting (a high-content cytological analysis technique) with convolutional neural networks (CNN) to accurately identify the differentiated and undifferentiated cells of iPSCs with high accuracy (F1-score ~0.98) [105] (Table 3). This study demonstrates the potential of AI-driven image analysis technology, providing a rapid, efficient, and non-destructive method in stem cell quality control. In future batch production of stem cells, this method can also ensure stem cell purity and consistency between batches, which is crucial for preclinical research and clinical translation. In addition to differentiating between stem cells and non-stem cells, it is equally important to exclude those stem cells that are aging. Ren et al. combined the Swin Transformer and group normalization techniques to develop a faster region-CNN detection model [106]. This model reduced the identification of senescent cells from the previous 10–12 h of SA-β-gal staining to just a few seconds, while maintaining high accuracy and reducing the computational complexity and memory usage of the model, achieving a mean average precision of 83.5% (Table 3).

When loading stem cells with nanomaterials, the application of hydrogels and topological structures alters the microenvironment of the stem cells, making the prediction of differentiation even more difficult. Dhaliwal et al. developed an algorithm that combined high-content image analysis and ML. By analyzing the three-dimensional characteristics of the nuclear splicing factor SC-35, it was possible to predict the 14-day differentiation outcome of the stem cells after only 72 h of implantation in 3D biomaterials [107]. Although the work focuses on the osteogenic and adipogenic differentiation of human MSCs, this strategy can be applied to NSCs or iPSCs, enabling early prediction with high precision (88%) and sensitivity (83%) (Table 3). By defining the AI-readable features of cell markers related to “differentiation into dopaminergic neurons” or “synaptic maturation”, such as neurite outgrowth morphology and the expression intensity and spatial distribution patterns of early neuronal markers like βIII-tubulin, we can predict the outcome at an early stage of differentiation. Similarly, the AI model can learn the cell characteristics related to the risk of tumor formation, thereby reducing the risk of tumor formation after transplantation.

How can we truly understand the underlying molecular mechanisms that control the behavior of stem cells? Reconstructing an accurate gene regulatory network (GRN) from single-cell RNA sequencing (scRNA-seq) data is currently a commonly used approach. However, inferring the GRN faces significant computational challenges, compounded by inherent technical limitations of scRNA-seq, such as dropout events and data sparsity. These issues can obscure true biological signals and adversely affect the reliability of AI-based predictions. In response, advanced computational frameworks have been developed. For instance, Wei et al. developed the gene regulatory network inference model Graph Attention Encoder-Decoder with Graph Random Walk Network (GAEDGRN) framework, which identified key genes using an enhanced PageRank algorithm that considers the degree of external connections. It can accurately identify key regulatory factors such as NANOG and TEAD4 and their target genes in human ESCs, demonstrating superior performance (AUROC: 0.905) compared to other benchmark methods [108] (Table 3). If we design a nano-enhanced stem cell using AI and observe its therapeutic phenotype, we can use GAEDGRN to interpret the mechanism of transplanted stem cells.

### In vivo tracking

The combination of nanomaterials with AI in the tracking of stem cell differentiation is truly remarkable. Stepanov et al. developed a method for tracing iPSCs called Live-cell Microscopic Imaging of Epigenetic Landscapes (LiveMIEL), a live-cell imaging platform that quantifies epigenetic dynamics [109]. Based on the chromatin structural domain of the MPP8 protein that can specifically bind to the H3K9me3 histone in living cells, they designed a gene-encoded epigenetic nanofluorescent probe. More importantly, the researchers combined the obtained complex fluorescence images with ML algorithms. Through principal component analysis and clustering of these multidimensional data, they successfully digitized and quantified the dynamic changes in the H3K9me3 epigenetic landscape during the differentiation of iPSCs into neurons. This work integrates nanoprobes, live-cell imaging, and AI, marking a leap from static, endpoint-based detection to the dynamic decoding of nanoscale biological processes within living cells. Among various ML methods, determining which one to use for predicting the differentiation performance of stem cells poses a challenge. Geng et al. compared the accuracy of Hierarchical Clustering Analysis, Logistic Regression Model, Linear Discriminant Analysis, and ANN in predicting the differentiation rate of human MSCs [110]. As a result, the accuracy of the ANN was almost completely consistent with the traditional immunocytochemical method.

In addition to the differentiation state, stem cell division also requires tracking. The division of stem cells can be classified into symmetric division and asymmetric division. Symmetric division refers to the division into two identical stem cells, while asymmetric division indicates the division into one stem cell and one differentiated cell. To achieve real-time tracking of the trend of stem cell division and lineage differentiation, Zhou et al. developed a comprehensive system integrating unmarked fluorescence lifetime imaging microscopy, feature extraction, and ML. Using ML, they screened out the lineage differentiation features of hematopoietic stem cells (HSCs) from the metabolic optical biomarkers feature library. In addition, they imaged the endogenous metabolic coenzymes NAD(P)H and FAD to quantify the division type of individual cells. With this tool, we can not only achieve continuous dynamic tracking of the differentiation process of HSCs in vitro, but also distinguish the division types within one hour after cell division.

### Post-transplant management

The value of AI lies not only in optimizing stem cell aspects but also in achieving precise prediction and personalized management of treatment outcomes. Asteris et al. developed an AI model to predict the long-term survival outcomes of patients undergoing allogeneic HSC transplantation (allo-HSCT). This algorithm intelligently selected and ranked 7 key parameters (including age, disease type, disease stage, creatinine level on the second day after transplantation, platelet implantation, and acute and chronic graft-versus-host disease) with the greatest influence from 18 potential clinical parameters, thereby constructing a model with an accuracy rate of up to 93.26% [111] (Table 3). This underscores the power of AI in leveraging structured clinical data.

However, multimodal biomarkers encompassing imaging, biochemical, and electrophysiological data are increasingly critical for predictive precision and personalized management [112]. For NDs, AI-driven frameworks that combine neuroimaging, cerebrospinal fluid profiles, and digital data can provide a more comprehensive and dynamic monitoring of post-transplant recovery and complications [112, 113]. Specifically, imaging techniques such as MRI and PET can track graft survival and neuroinflammation. Serial biochemical profiling, including cytokines and neurodegeneration-specific blood biomarkers, offers insights into systemic and cellular responses. Electrophysiological signals, such as EEG, can monitor functional network recovery. Together, they provide a real-time, multidimensional feedback of therapeutic response and adverse events. Reyes et al. have utilized multimodal AI models to identify AD subtypes at early stages, outperforming single-modality approaches [114]. Although no AI model has been developed to predict the long-term survival rate of patients with NDs, the approach is poised to enhance clinical decision-making, ultimately aiming to improve survival rates and quality of life.

In short, the deep integration of AI technology marks a profound bridge in the treatment of NDs with nano-engineered stem cells. It enhances our capabilities in the design of nanomaterials, the prediction of stem cell fate, post-transplantation tracking, and clinical efficacy management. This is conducive to promoting the transformation of nanomedicine towards data-driven, precise design, real-time monitoring, and personalized intervention models. However, the widespread application of current AI models still relies on the accumulation of high-quality big data and the deepening of algorithm interpretability. In the future, with the further integration of multi-omics data and dynamic imaging information, AI is expected to accelerate the transformation of safe and efficient stem cell nanotherapies from the laboratory to clinical practice.

**Table 3:** Performance comparison of AI models in optimizing nano-engineered stem cell therapy

Refs	AI model	Dataset type	Primary task	Results and key performance metrics
[99]	Equation Learning with Bayesian-Informed Neural Network	Time-series flow cytometry data	Infer kinetic models of nanoparticle-cell interactions.	High reliability in recovering correct model forms Focusing on interpretability
[100]	Artificial Neural Network	Experimental data	Predict PLGA nanoparticle size. Classify the size distribution quality.	R² = 0.9734 Root Mean Square Error (RMSE) = 43.588 nm Accuracy = 100% in identifying good and bad distributions
[102]	Random Forest	Literature-extracted dataset	Predict the percentage composition of functional protein classes in the nanoparticle corona	R² > 0.75 RMSE < 5%
[104]	Genetic Algorithm-Partial Least Squares	Experimental data	Predict nanoparticle zeta potential (ζ) from core, coating, and corona descriptors	R² = 0.957 RMSE of Prediction = 7.331
[105]	Convolutional Neural Network	Cell Painting microscopy images of iPSC co-cultures	Classify cell types (neurons and progenitors) in iPSC co-cultures	F1-score: ~0.98
[106]	Swin Transformer-based Giga-pixel Fast R-CNN with Graph Normalization	Microscopy images of iPSCs	Detect and classify senescent vs. young induced pluripotent mesenchymal stem cells for drug screening	Mean average precision = 83.5% F1-score: 90.5% (senescent), 92.1% (young)
[107]	J48 Decision Tree (with Haralick texture features)	3D nuclear organization images of human MSCs	Predict human mesenchymal stem cell differentiation lineage (osteogenic/adipogenic)	Sensitivity = 83% Precision = 88%
[108]	Graph Attention Encoder-Decoder with Graph Random Walk Network	ScRNA-seq data	Infer directed Gene Regulatory Networks	Average Area Under the Receiver Operating Characteristic Curve (AUROC) = 0.905 Area Under the Precision-Recall Curve (AUPRC) = 0.758
[109]	Principal Component Analysis & Expectation-Maximization Clustering (LiveMIEL)	Live-cell imaging data	Quantify and cluster dynamic H3K9me3 epigenetic patterns during live iPSC differentiation	Pearson’s *R* = 0.81 Analysis of 98 image features clustered differentiation into 3 stages (0, 1–2, 3–4 days) with cluster tendency (Hopkins statistic H = 0.28)
[110]	Comparative Analysis: Artificial Neural Network. Logistic Regression Model, Linear Discriminant Analysis	Label-free Raman spectra	Predict neural stem cell differentiation rate	Artificial Neural Network predictions were nearly identical to immunocytochemistry gold-standard rates. Logistic Regression Model and Linear Discriminant Analysis showed slightly lower but consistent accuracy, validating the ANN’s superiority for this non-linear task
[111]	Data Ensemble Refinement Greedy Algorithm (with Extra Trees Classifier)	Clinical data	Predict long-term survival post-allogeneic hematopoietic stem cell transplantation	Accuracy = 93.26%

## Challenges and future perspectives

### Foundational technical bottlenecks

Although AI relies on a large amount of data, experimental data in the field of stem cell nanomedicine is lacking large-scale and well-labeled datasets [115]. Generating high-quality labeled data, such as manually delineating cell boundaries or identifying the interactions of nanomaterials, remains a labor-intensive task that requires expertise [116]. The scarcity of such data limits the training of advanced models and leads to overfitting, where the algorithm performs well on the training data but fails to generalize to new conditions [117]. Beyond data scarcity, batch-to-batch variability in nanomaterial synthesis and stem cell culture critically affects AI model generalization [118, 119]. Minor variations in production or culture conditions can significantly alter key properties like nanoparticle size. If trained on limited batches, models may reduce their generalizability to new batches. To overcome this, training datasets must intentionally include diverse multi-batch and multi-protocol data. Incorporating advanced techniques like domain adaptation or physics-informed AI is also key to developing robust, clinically translatable models [120, 121]. In addition to the data volume, the diversity of the data constitutes another obstacle. The interdisciplinary research in AI-guided nanoengineered stem cells integrates information from clinical information, imaging, genomics, nanomaterial properties, and stem cells. Differences in experimental protocols, equipment, and biological environments further exacerbate this inconsistency [122]. Without standardized and unified data representation methods, it is scarcely possible to receive comprehensive results from multiple studies.

Another critical issue is data availability. Training data for AI models primarily originates from private or institution-specific studies. Due to proprietary interests, ethical constraints, and non-standardized sharing protocols, these data are seldom accessible, limiting the model’s training. To harness the value of these data while preserving privacy, Federated Learning (FL) is emerging as a solution. FL is a distributed ML method that allows multiple participants to collaboratively train the model without exchanging any raw data, thereby protecting privacy. Notably, recent studies have shown that FL has been successfully applied in the research of NDs, such as predicting the progression from mild cognitive impairment to AD and using multi-institutional data to improve PD drug development [123, 124]. Applying FL to a multi-institutional dataset containing nanobiomaterial parameters, stem cell responses, and patient outcomes will drive the next generation of AI-driven design tools.

Furthermore, the reproducibility of AI-based stem cell analysis, particularly in tracking, is heavily impacted by different image acquisition conditions. Various microscope hardware, imaging protocols, lighting, magnification, and sample preparation can introduce substantial heterogeneity into the training data [125, 126]. A model trained on images from a specific environment may fail to generalize to data acquired under different conditions, undermining the robustness and broad applicability [127]. This challenge necessitates the development of standardized imaging protocols and reliable AI models to account for and mitigate variations.

The reliance on static AI models presents another fundamental methodological bottleneck. These models operate on fixed data, inherently limited in exploring beyond known parameter spaces. Dynamic models such as reinforcement learning and generative models are therefore critical for autonomous design. Reinforcement learning navigates the complex multiple parameters through iterative simulations, dynamically balancing stem cell targeting, therapeutic delivery, and biocompatibility [128]. Generative models learn the existing successful nano-enhanced stem cell examples and then propose a novel design [24].

Simply training a model on existing data is not enough. Rigorous validation is essential for predicting differentiation outcomes and preventing overfitting [129, 130]. Two key methods are commonly used in models built to identify cell types or predict functional outcomes based on morphological or dynamic features [129–131]. k-fold cross-validation can assess model stability and performance on different data subsets [131, 132]. Another is blind testing on completely independent datasets. In a word, this validation step verifies the predictive ability in real-world and heterogeneous scenarios [133].

Beyond data and privacy considerations, developing and training complex models demand significant computational and hardware resources [134, 135]. Generating sufficient high-quality training data in experimental fields like materials science or biomedicine costs much in terms of time, specialized expertise, and materials [136]. A major hardware bottleneck lies in the widely used backpropagation algorithm [137, 138]. Its need for frequent data transfer between memory and processing units makes training slow and expensive on conventional hardware [135, 139, 140]. This drives the search for more energy-efficient and scalable computing hardware. Although neuromorphic computing and cloud computing are emerging, their accessibility, cost, and integration are still problems [141, 142]. Model optimization, efficient algorithms, and novel hardware will accelerate AI-driven nano-engineered stem cell therapy for NDs.

The interpretability of AI models remains a significant challenge. While simple ML models permit some interpretability through feature importance analysis, their complex counterparts often function as “black boxes”, impeding the elucidation of their prediction rationale [143–145]. Improving model transparency is not only crucial for verification but also significant for proposing new biological hypotheses [146]. Recently, experts in the field of materials science have provided us with a blueprint. When traditional density functional theory descriptors were unable to explain the catalytic performance observed in experiments, Suvarna et al. developed an Explainable AI (XAI) model [147]. By combining the decision tree model CRISP and the RF model SPIRO, they clarified the complex relationship of AI-guided electrocatalysts. These comprehensive models were verified through experiments, with an overall accuracy rate exceeding 80%, achieving the transition from predictive AI to XAI.

### Clinical translational problems

As AI-driven nano-enhanced stem cell therapy combines stem cells, nanomaterials, and AI, its translation from bench to bedside introduces complex regulatory and ethical challenges [148–150].

The long-term safety of nanoparticles persisting in neural tissue requires careful evaluation [6]. For example, while SPIO labeling enables excellent MRI tracking, internalization can induce iron overload and ferroptosis in MSCs, potentially compromising their viability and paracrine function [61, 62]. Moreover, dose-dependent cytotoxicity and apoptosis have been observed in human neural progenitor cells and rat brain tissue upon exposure to gold nanoparticles [151]. To address this, developing biodegradable or biocompatibility nanomaterials is important, such as advanced core-shell magnetoelectric nanoparticles [152].

The potential impact of external guidance systems on the neural circuitry and BBB should be considered. A recent preclinical study indicates that time-varying magnetic fields can induce electrical currents in neural tissue, posing neuromodulation and disrupting local electrophysiology [153]. Concurrently, excessive ultrasound energy can transform reversible BBB opening into harmful microhemorrhages and inflammation [154]. To prevent adverse neural or vascular events, precise physical parameter limits must be paired with live multimodal feedback.

Regulatory and manufacturing-commercialization are significant challenges. Clear classification and a pathway are lacking. Agencies may categorize it as an advanced therapy medicinal product (ATMP) with borderline status [155]. Thus, a novel framework is required to concurrently evaluate safety, biocompatibility, and reliability. Besides, for a product comprising stem cells, nanomaterials, and AI, the U.S. Food and Drug Administration or European Medicines Agency may classify it as a combination product [156, 157]. This necessitates a coordinated review process across different centers, which involves integrating distinct and even conflicting guidelines for safety, efficacy, and quality. On the manufacturing and commercialization front, three major challenges arise. Firstly, Good Manufacturing Practice for personalized production requires a tightly controlled framework, with quality control shifting from batch release to real-time Process Analytical Technology verification [157]. Secondly, the manufacturing of bespoke nanomaterials and their integration with stem cells remains resource-intensive, although AI and automation can streamline design. Thirdly, trained AI, specialized personnel, and small-batch GMP manufacturing require high costs. Therefore, superior clinical outcomes are essential for getting more support.

AI-guided in silico immunogenicity represents a favorable choice for safety. Traditional experimental screening for unintended immune activation is costly. Fortunately, AI is revolutionizing the predictive assessment of nanomaterial-immune system interactions from sequence and design data. For instance, DL models and large language models are now being employed to predict T-cell immunogenicity scores [158]. Furthermore, computational frameworks optimize nanoparticle design while assessing immune activation in silico to evaluate immune activation risks, as demonstrated in the mRNA vaccine delivery [159]. These AI-powered in silico tools can be embedded within nano-engineered stem cell design, minimizing immunogenicity risks in early development stage.

Adapted clinical trial design and heightened safety-accountability are required. In trial design, validating an AI model trained on retrospective data in prospective trials and defining endpoints for a dynamic therapy are major hurdles [160, 161]. The locked algorithm requirement in traditional trials conflicts with the potential need for iterative AI model updates based on new patient data. Furthermore, safety and accountability extend to the AI component. Algorithmic errors or biases could lead to toxic or suboptimal nanomaterials [162]. Establishing clear accountability among biologists, material scientists, and AI developers is crucial for adverse event investigation. XAI is thus a safety and regulatory necessity, not just a scientific desire [163]. Moreover, data governance and ethical imperatives are of great concern. Rapid development relies on large multimodal datasets, raising issues of data privacy and security, especially in FL setups [164–166]. Informed consent must transparently communicate the roles of AI and nanomaterials. Algorithmic fairness must be audited to ensure the AI does not exacerbate health disparities by performing poorly on underrepresented groups.

## Conclusion

The limited regenerative capacity of the central nervous system is a formidable barrier that traditional drug treatments for NDs cannot overcome. Recently, stem cells emerged as a solution. However, the harsh inflammatory microenvironment, imprecise delivery, and untargeted differentiation have slowed the clinical translation of stem cells. This review combines two transformative technologies, nanomaterials and AI, to break through these obstacles and develop a new generation of intelligent personalized treatment plans for NDs based on stem cells. Nanomaterials are responsible for the protection and positioning of stem cells. AI is good at processing massive data to optimize the design of nano-enhanced stem cells and monitor the post-transplantation situation. This partnership transcends simple enhancement, enabling unprecedented levels of prediction, control, and personalization in regenerative neurology. The current challenges of scarce data and model interpretability are not obstacles but the next frontier of research. This requires the development of generative AI models, interpretable AI, and causal inference networks to unveil the mystery of the “black box”. To realize this vision, a phased roadmap is critical. In the near term, the focus is on developing and validating foundational AI tools to predict nanoparticle-cell interactions, protein corona formation, and stem cell differentiation. In the mid-term, these models will be integrated into multi-parameter in silico platforms for designing optimized nano-engineered stem cell therapies. The long-term goal is the clinical deployment of an adaptive closed-loop system, integrating patient-specific data, AI-driven design, automated manufacturing, and real-time monitoring to dynamically personalize therapy for NDs. Ultimately, we envision a patient-specific digital twin for NDs, an AI-driven and dynamic virtual replica of an individual [167–170]. By assimilating multimodal data and simulating disease progression, this twin would enable in silico optimization and personalized forecasting of nano-enhanced stem cell therapy [171, 172]. The combination of AI, nanotechnology, and stem cells will bring hope to millions of people worldwide suffering from NDs.

## Data Availability

No datasets were generated or analysed during the current study.

## Abbreviations

NDs: Neurodegenerative diseases
AI: Artificial Intelligence
ML: Machine learning
DL: Deep learning
PD: Parkinson’s disease
AD: Alzheimer’s disease
ALS: Amyotrophic lateral sclerosis
ESCs: Embryonic stem cells
iPSCs: Induced pluripotent stem cells
MSCs: Mesenchymal stem cells
NSCs: Neural stem cells
CSF: Cerebrospinal fluid
ROS: Reactive oxygen species
BBB: Blood-brain barrier
SPIO: Superparamagnetic iron oxide
VCAM-1: Vascular cell adhesion molecule 1
RA: Retinoic acid
siSOX9: Small interfering RNA targeting SOX9
MRI: Magnetic resonance imaging
UCNPs: Upconversion nanoparticles
NIR: Near-infrared
PAI: Photoacoustic imaging
ANN: Artificial neural network
RF: Random forest
CNN: Convolutional neural networks
GRN: Gene regulatory network
ScRNA-seq: Single-cell RNA sequencing
GAEDGRN: Graph Attention Encoder-Decoder with Graph Random Walk Network
LiveMIEL: Live-cell Microscopic Imaging of Epigenetic Landscapes
HSCs: Hematopoietic stem cells
FL: Federated learning
XAI: Explainable AI
